# Conceptualizing Acceptance and Knowledge as Process Variables in Internet-Delivered and Therapist-Supported Cognitive Behavioral Therapy and Acceptance and Commitment Therapy in Primary Care for Insomnia: Pilot Feasibility and Process-Oriented Randomized Controlled Trial

**DOI:** 10.2196/81285

**Published:** 2026-05-21

**Authors:** Anna Caroline Larsson, Susanna Jernelöv, Viktor Kaldo, Kerstin Blom, Sandra Weineland

**Affiliations:** 1 General Practice/Family Medicine, School of Public Health and Community Medicine, Institute of Medicine, Sahlgrenska Academy, University of Gothenburg, Box 400, Gothenburg, 405 30, Sweden, 46 (0)31 786 00 00; 2 Research, Development, Education and Innovation, Primary Health Care, Region Västra Götaland, Borås, Sweden; 3 Centre for Psychiatry Research, Department of Clinical Neuroscience, Karolinska Institutet, & Stockholm Health Care Services, Region Stockholm, Sweden; 4 Division of Psychology, Department of Clinical Neuroscience, Karolinska Institutet, Stockholm, Sweden; 5 Department of Psychology, Faculty of Health and Life Sciences, Linnaeus University, Växjö, Sweden; 6 Department of Psychology, University of Gothenburg, Gothenburg, Sweden

**Keywords:** acceptance, acceptance and commitment therapy, internet-delivered acceptance and commitment therapy, iACT, internet-delivered cognitive behavioral therapy, iCBT, insomnia, internet intervention, knowledge, primary care

## Abstract

**Background:**

Internet-based interventions for insomnia show promise, but understanding the process variables, such as knowledge acquisition and psychological acceptance, is crucial for enhancing digital adherence and clinical effectiveness.

**Objective:**

This study aimed to evaluate the feasibility, adherence, and preliminary clinical signals of 2 therapist-assisted interventions—internet-delivered cognitive behavioral therapy (iCBT) and internet-delivered acceptance and commitment therapy (iACT)—for insomnia in a primary care setting.

**Methods:**

This was a pilot randomized controlled trial. Adults seeking help for insomnia (n=18) were recruited via primary care and randomized to either a 5-module iCBT or iACT program delivered via a secure digital platform with weekly therapist feedback. Blinding of participants and therapists was not possible due to the nature of the interventions. Primary outcomes included the Insomnia Severity Index; secondary outcomes included the 9-item Patient Health Questionnaire, 7-item Generalized Anxiety Disorder, and WHO Disability Assessment Schedule. A novel sleep knowledge test was used as a process variable. The data were analyzed using split-plot analyses of variance (intention-to-treat or last observation carried forward and complete case analysis) and nonparametric Friedman and Kruskal-Wallis tests.

**Results:**

A total of 18 participants were randomized (iCBT: n=9; iACT: n=9). High attrition was observed, with only 33.3% (n=3) of iCBT and 55.6% (n=5) of iACT participants completing all modules. The iACT group demonstrated a significant within-group reduction in insomnia severity (*P*=.01, Friedman test), whereas iCBT results were nonsignificant (*P*=.10, Friedman test). No significant between-group differences were found for any clinical or process variables. Participants rated both treatments as credible (Credibility/Expectancy Questionnaire scores remained stable), though qualitative feedback indicated a need for more flexible, less burdensome content.

**Conclusions:**

This pilot study demonstrates that while internet-delivered insomnia treatments are feasible and credible in primary care, high attrition remains a significant barrier. Preliminary signals suggest that iACT may be a viable alternative to iCBT, potentially offering better adherence. Larger, fully powered pilot randomized controlled trials (estimated N=404) with refined recruitment and automated retention strategies are required to determine definitive comparative efficacy and the mediating role of sleep knowledge and acceptance.

## Introduction

### Background

Sleep is essential for both physical health and mental well-being. However, insomnia is one of the most common sleep disorders in modern society, characterized by persistent difficulties with sleep quantity or quality [1]. In Sweden, approximately 10% of the population meets the criteria for insomnia [2]. Given this high prevalence and the associated public health burden, it is crucial to develop accessible, nonpharmacological treatments that can reach a broad population, especially considering that over 80% of sleep medication users express a preference for psychological interventions [3].

Cognitive behavioral therapy for insomnia is the gold-standard treatment, showing strong evidence for reducing insomnia severity [4]. Grounded in cognitive restructuring and behavioral modification, cognitive behavioral therapy for insomnia focuses on altering dysfunctional thoughts and sleep-related behaviors [5,6]. However, the demanding nature of certain techniques, such as sleep restriction, can exacerbate fatigue and lead to adherence challenges [7,8]. Acceptance and commitment therapy (ACT) has emerged as a promising third-wave alternative. Rather than focusing on the direct modification of sleep-related thoughts, ACT aims to enhance psychological flexibility by promoting mindful awareness and the acceptance of internal distress [9,10]. By reducing anxiety about wakefulness, ACT helps individuals cultivate a more flexible approach to sleep-related difficulties [11,12].

The evidence base for internet-delivered acceptance and commitment therapy (iACT) remains limited compared to internet-delivered cognitive behavioral therapy (iCBT). Preliminary head-to-head trials suggest that while iCBT may lead to more rapid initial improvements, the effects of iACT appear to consolidate over time, with both groups reaching comparable outcomes at follow-up [13]. Furthermore, emerging research suggests that psychological acceptance functions as a key driver of clinical improvement [14]. Despite these insights, a significant research gap remains: there is a lack of comparative studies focusing on the underlying processes of change within a routine primary care setting.

In the current landscape of digital psychotherapy, iCBT and iACT represent 2 distinct theoretical pathways. iCBT emphasizes knowledge acquisition and the subsequent application of behavioral strategies, whereas iACT prioritizes the process of acceptance. In this trial, these constructs are evaluated as process variables rather than formal causal mechanisms. Given the pilot nature of the study, the focus is on observing how these variables shift during a condensed intervention, an essential step for improving clinical feasibility, as shorter digital protocols have been shown to maintain efficacy while potentially reducing the high attrition rates seen in long-form treatments [15,16].

### Aim and Research Questions

The primary aim of this pilot RCT was to assess the feasibility of the study protocol within a routine primary care setting, specifically regarding recruitment rates, treatment adherence, and assessment procedures. Secondary aims were to explore preliminary treatment effects and examine sleep-related knowledge and acceptance as potential process variables in therapist-supported iCBT and iACT for insomnia.

We hypothesized that both groups would show improvements in insomnia severity and psychological symptoms. Furthermore, we expected differential changes in process variables: the iCBT group was hypothesized to show greater gains in sleep-related knowledge, whereas the iACT group was expected to demonstrate greater improvements in psychological flexibility and sleep-related acceptance.

The research questions can be concretized as follows:

1. Feasibility outcomes: Is it feasible to conduct a larger-scale randomized controlled trial (RCT) with respect to participant recruitment, intervention design, and assessment methods? Feasibility was evaluated using data on recruitment, treatment adherence, the Credibility/Expectancy Questionnaire (CEQ), as well as participant feedback on treatment attitudes.
2. Process outcomes: How do the interventions differ in terms of responses on the sleep knowledge test*,* psychological flexibility as measured by the Swedish Acceptance and Action Questionnaire (SAAQ), and sleep-related acceptance as measured by the Sleep Problem Acceptance Questionnaire (SPAQ)?
3. Primary outcomes: What preliminary effect does iCBT have on insomnia compared to iACT, as measured by the Insomnia Severity Index (ISI)?
4. Secondary outcomes: What are the preliminary effects of the interventions on depressive symptoms, anxiety, and functional impairment as measured by the 9-item Patient Health Questionnaire (PHQ-9), the 7-item Generalized Anxiety Disorder scale (GAD-7), and the WHO Disability Assessment Schedule (WHODAS), respectively?

## Methods

### Trial Design and Registration

This pilot RCT was conducted and reported in accordance with the CONSORT-EHEALTH (Consolidated Standards of Reporting Trials of Electronic and Mobile Health Applications and Online Telehealth) checklist (V 1.6.1) [17] (Checklist 1) and the CONSORT (Consolidated Standards of Reporting Trials) 2010 extension for pilot and feasibility trials [18]. Although the trial was not preregistered, the project is registered in the Researchweb database of the Västra Götaland Region (VGR) (project number 272866). The full study protocol is available from the corresponding author upon reasonable request.

### Study Design

This is a pilot RCT conducted at 16 primary health clinics in the primary care in VGR in Sweden. Patients of 18 years of age with insomnia who seek or are referred to the clinics were invited to participate. Participation required informed consent from the patient. The study used a randomized controlled design. Patients were allocated using a pregenerated and concealed randomization sequence. The interventions were presented as 2 distinct treatments derived from a combined original protocol. Thus, neither was framed as a control group. As the specific names of the interventions were disclosed during the consent process, participant preferences were possible. In 8 cases, patients declined participation specifically because they preferred receiving the combined original intervention rather than being randomized to a single treatment arm.

To assess the feasibility of these interventions in primary care, this study integrated clinical data with theoretical insights to evaluate a modified treatment format. A central objective was to preliminarily explore the roles of knowledge and acceptance within digital treatments, providing an initial basis for understanding process variables in a primary care context. To investigate this, an existing 8-module internet-delivered intervention was adapted into 2 concise 5-module programs (iCBT and iACT) to evaluate if a shortened format could maintain engagement. By testing this streamlined approach and new assessment tools, the study aims to identify ways to enhance treatment efficiency and reduce participant dropout in future large-scale trials.

Before initiating the recruitment, a randomization sequence was generated all at once by a research assistant using Randomizer.org and implemented by the research leader. The tool was configured to allow duplicate values, meaning that numbers were not required to be unique and were presented in an unsorted, random order. Simple randomization was used without blocking or stratification, and the sequence did not ensure equal group sizes or balance across treatment arms. This study was conducted in accordance with the CONSORT-EHEALTH checklist (V 1.6.1) [17] (Checklist 1) and adapted to the CONSORT extension for randomized pilot and feasibility trials [18].

### Participants

Patients were referred by 16 primary health clinics through a standardized digital referral system. Initial assessment interviews were conducted by therapists at the research unit to determine study eligibility. The participants were recruited among adults seeking help for sleep problems through primary care services. Inclusion criteria required individuals to be aged 18 years or older, exhibiting sleep-related issues, having access to a computer or smartphone with internet connectivity, and being able to read and write in Swedish. A total of 18 participants were enrolled in the study, with 9 assigned to iCBT and 9 to iACT. Demographic characteristics of the participants are presented in Table 1.

In the iCBT-group, the mean age was 39.22 (SD 15.43) years, whereas the mean age of the iACT-group was 36.67 (SD 11.25) years.

**Table 1.** Baseline demographic characteristics of participants in the internet-delivered cognitive behavioral therapy (iCBT) and internet-delivered acceptance and commitment therapy (iACT) groups (n=18).

Variable	iCBT (n=9), n (%)	iACT (n=9), n (%)
Age (y)		
18‐25	1 (11)	3 (33)
26‐35	4 (44)	0 (0)
36‐45	2 (22)	1 (11)
46‐55	1 (11)	4 (44)
56‐65	1 (11)	1 (11)
Gender		
Female	5 (55)	6 (66)
Male	4 (44)	3 (33)
Other	0 (0)	0 (0)
Occupation		
Employed full-time	4 (44)	6 (66)
Employed part-time	0 (0)	1 (11)
Night-shift work	0 (0)	0 (0)
Job applicant	1 (11)	0 (0)
Student	0 (0)	2 (22)
Sick leave	3 (33)	0 (0)

### Ethical Considerations

The study was approved by the Regional Ethics Committee in Gothenburg (Dnr: 767‐18) and was conducted in accordance with the Declaration of Helsinki. All participants provided informed consent prior to inclusion. Digital data were handled in accordance with the General Data Protection Regulation, ensuring participant confidentiality through pseudonymization.

### Procedure

Participant recruitment took place within the regular patient flow. Following the referrals from the 16 clinics, study therapists conducted clinical assessment interviews via video call or telephone using the structured Mini International Neuropsychiatric Interview (MINI) [19] to establish insomnia diagnoses and comorbid diagnoses.

The inclusion criteria were fulfilling the diagnostic criteria for Insomnia Disorder according to the *Diagnostic and Statistical Manual of Mental Disorders, Fifth Edition* (*DSM-5*) and the *International Classification of Diseases, 10th Revision* (*ICD-10*; confirmed by ISI and clinical interview), having access to an internet-connected device, a Swedish BankID, and sufficient eHealth literacy. Exclusion criteria were a neuropsychiatric diagnosis, intellectual disability, bipolar disorder, substance abuse, or insufficient proficiency in Swedish. Both current and previous suicide risk were grounds for exclusion.

Following the interview, eligible patients were contacted by the research leader, who provided detailed verbal and written information about the 5-module study format in comparison to the standard 8-module iCBT. To ensure the integrity of the process, participants were not informed of their specific group assignment until after providing formal written informed consent.

Initial verbal consent was obtained prior to randomization. Patients who declined were then offered standard 8-module iCBT. The patients who provided verbal consent were assigned by the research leader to either iCBT or iACT using the pregenerated sequence. Due to the nature of the psychological interventions, no blinding was performed. Participants accessed the platform via BankID and provided formal written consent (Multimedia Appendix 1) upon their first login. Once the trial had started, no major changes to the methods were made. To ensure a balanced distribution of therapist effects, the 18 participants and the 2 treatment arms were distributed equally within the caseloads of the 3 participating therapists.

### Data Management and Security

Treatment was delivered via the Stöd och behandling (SOB) platform, where login credentials were maintained separately from patient responses. All study-related questionnaires and informed consent forms were administered digitally through the secure survey tool esMaker. In compliance with the General Data Protection Regulation, all personal identification data and code lists were stored separately from the study data on secure servers within the VGR infrastructure. Code lists and digital consents were managed by the central iCBT unit (EBBA-Team) in area V7, Sjuhärad. Upon the completion of the study, pseudonymized datasets were transferred to the Primary Care Research and Development Unit in Södra Älvsborg for analysis. To ensure participant anonymity, coded identifiers were used. The corresponding code key was stored securely and was accessible only to 2 lead researchers.

### Interventions

The interventions were delivered through the national Swedish eHealth platform SOB, managed by Inera AB. Access required Swedish BankID via the national patient portal “1177.” The treatment was provided and clinically supervised by Närhälsan, Region Västra Götaland. The core iCBT protocols were originally developed by licensed psychologist Lars Ström, PhD (Livanda AB) [20].

For the current trial, the original 8-module iCBT program was adapted into 2 distinct 5-module interventions (iCBT and iACT). This adaptation was led by the first author (ACL) in collaboration with Lars Ström and under the supervision of co-author SW. The final content underwent a quality review by sleep experts at the Karolinska Institute (coauthors SJ, VK, and KB).

Each program included psychoeducational content, videos, audio files, exercises, and symptom-tracking tools. Patients worked through 1 module per week and completed structured home assignments. Asynchronous therapist support was provided via the SOB platform by either a licensed psychologist or a PTP-psychologist (Praktisk Tjänstgöring för Psykologer), a psychology graduate completing a mandatory year of supervised practice for licensure under senior supervision. Therapists monitored progress and provided individualized feedback (typically one message per module). In cases of decreased engagement, a standardized follow-up protocol (digital messages, calls, and letters) was initiated. Brief synchronous telephone follow-ups occurred at midtreatment and posttreatment. A summary of the treatment modules is provided in Table 2.

**Table 2.** Summary of treatment modules and core therapeutic components for the internet-delivered cognitive behavioral therapy (iCBT) and internet-delivered acceptance and commitment therapy (iACT) interventions^a^.

Serial number	iCBT	iACT
1	Introduction Psychoeducation on sleep Diaphragmatic breathing Sleep restriction Optional: about CBT	What is important for you? The life compass Diaphragmatic breathing Optional: about ACT
2	Methods for improving sleep Psychoeducation on sleep Stimulus control Psychoeducation on sleep problems	Sleepy thoughts Mindfulness techniques for managing thoughts Basic body scan
3	Manage disturbing thoughts Psychoeducation on distressing thoughts Conditioned body scan	The thought machine Relational frame theory techniques and metaphors
4	Stress and sleep disturbances Psychoeducation on sleep disturbances Psychoeducation on stress behaviors	Mindfulness Mindfulness techniques for being present Quick body scan
5	Progress plan Plan for maintaining change	Conclusion Plan for maintaining change

a Both interventions were delivered over 5 weeks via a secure online platform. Modules were designed to be completed sequentially.

### Measures

Assessment and inclusion data were collected using the MINI [19] during the professional interview to determine differential diagnoses. The MINI includes modules for depression, suicidality, bipolarity, panic disorder, and other Axis I disorders, exhibiting acceptable validity and reliability [21]. The remaining data were collected online, and reporting followed the CHERRIES (Checklist for Reporting Results of Internet E-Surveys) [22].

Participants provided demographic information at pretreatment, including age, employment status, and duration of insomnia. Substance use was screened using the Alcohol Use Disorders Identification Test (AUDIT) [23] and the Drug Use Disorders Identification Test (DUDIT) [24].

Feasibility was evaluated based on participant recruitment rates, treatment adherence (operationalized as the number of modules initiated), and participant attitudes. Qualitative reasons for discontinuation were collected through short phone interviews at the time of dropout to identify potential barriers to the intervention design.

Treatment credibility and outcome expectations were assessed using the CEQ, a well-established instrument with demonstrated test-retest reliability [25,26]. These were measured at baseline and midtreatment to monitor shifts in participants’ attitudes. Additionally, posttreatment qualitative feedback was gathered using a questionnaire adapted from previous internet-based therapy research [27], assessing perceived knowledge gain, preferences for treatment format, and specific likes or dislikes regarding components such as sleep restriction and mindfulness.

To evaluate hypothesized mechanisms of change, psychological flexibility was assessed using the SAAQ [28], and acceptance of sleep-related difficulties was measured with the SPAQ [29,30]. Both were administered at pretreatment, midtreatment, and posttreatment. The sleep knowledge test (detailed below) was also used as a key process variable.

The primary outcome, insomnia severity, was measured using the ISI [31] at pretreatment, midtreatment, and posttreatment. The ISI is the standard self-assessment scale in insomnia research [32], demonstrating strong internal consistency and sensitivity to change [33]. Furthermore, the ISI has been specifically validated for use as a web-based measure, maintaining its psychometric integrity when administered online [34].

Secondary clinical outcomes included depression symptoms (PHQ-9) [35], measured at pretreatment and posttreatment, and anxiety symptoms (GAD-7) [36], measured at pretreatment, midtreatment, and posttreatment. Functional impairment related to health status was evaluated using the WHODAS [37,38] at pretreatment, midtreatment, and posttreatment.

To assess treatment engagement and curriculum mastery, a study-specific 30-item sleep knowledge test was used. The instrument was adapted from a measure originally developed by coauthor SW [27]. For the current trial, the adaptation and further development of the test were led by the first author (ACL) under the supervision of SW, and further refined in collaboration with sleep experts at Karolinska Institutet (coauthors SJ, VK, and KB) to ensure alignment with the 5-module intervention content.

The development followed an iterative process that included pilot testing among 19 health care professionals at a primary health center to ensure intelligibility and feasibility. This pilot group consisted of 8 general practitioners, 5 nurses and assistant nurses, 4 administrators, 1 physiotherapist, and 1 occupational therapist (1 participant did not report a profession). Initially consisting of 20 items, the test was reviewed by this group, who correctly answered an average of 13 items; the test was expanded to 30 items for the final trial to evaluate the full breadth of the curriculum.

Once the trial began, no major changes to the sleep knowledge test were made. Given its formative nature, where items represent distinct educational facts rather than a single latent psychometric construct, traditional measures of internal consistency (eg, Cronbach α) were interpreted with caution. Analysis focused instead on item-level performance and content validity. The version used in this trial represented an early stage of development, aimed at gathering preliminary data for further refinement. An English translation of the full test is provided in Multimedia Appendix 2.

### Sample Size and Attrition

The target sample size was 90 participants (45 per group), estimated to detect between-group differences of a moderate magnitude (*d*=0.35-0.40) with 80% statistical power and a 2-sided significance level of *α*=.05. Participants were recruited from 16 health care clinics using a standardized digital referral process. Interventions were delivered uniformly across sites to mitigate the risk of clustering effects by clinic.

### Data Analysis

Statistical analyses were performed using IBM SPSS Statistics (version 28; IBM Corp). The primary focus of this pilot study was on descriptive metrics, as the final sample size (n=18) did not provide sufficient power for stable, complex inferential testing. By prioritizing a descriptive approach, we aimed to provide a transparent overview of feasibility and preliminary trends while avoiding the risk of unstable statistical inferences. Consequently, these results are intended to inform the design of future, adequately powered trials rather than establish definitive mechanistic conclusions. No subgroup or adjusted analyses were performed due to the pilot nature and small sample size.

To maximize data usefulness, we used an observed case analysis that included all available measurements at each time point, irrespective of treatment completion. To address missing data and ensure the robustness of our findings, we also conducted sensitivity analyses using last observation carried forward (LOCF) and complete case analysis (CCA). While LOCF facilitates the inclusion of dropout data, a critical component for feasibility studies, we acknowledge its potential for bias in final efficacy evaluations. Conversely, CCA was used under the assumption that data were missing completely at random [39].

For data meeting parametric requirements, split-plot ANOVA and independent samples 2-tailed *t* tests were used to investigate within-group and between-group variance across the measurement occasions (pretreatment, midtreatment, and posttreatment). To cross-validate these findings and address nonparametric data distributions, Friedman tests for repeated within-group measures and Kruskal-Wallis tests for between-group comparisons were also conducted. Effect sizes (Cohen *d*) and 95% CIs are reported for primary outcomes to indicate the magnitude of change and facilitate future power calculations.

Following a preplanned protocol, the Likert scale questions in the CEQ (1-9), the mid-range scores (4-6) were merged into a single neutral category to facilitate comparison between high and low expectancy groups [40]. For the sleep knowledge test, the analysis prioritized a descriptive exploration of content mastery rather than formal psychometric evaluations, such as floor or ceiling effects, which were deemed statistically premature at this stage. Finally, treatment adherence was analyzed descriptively using frequencies and percentages, while secondary clinical outcomes (PHQ-9, GAD-7, and WHODAS) were analyzed using both LOCF and CCA to explore preliminary clinical signals in comorbid symptoms.

## Results

### Participant Recruitment and Flow

**Figure 1.**
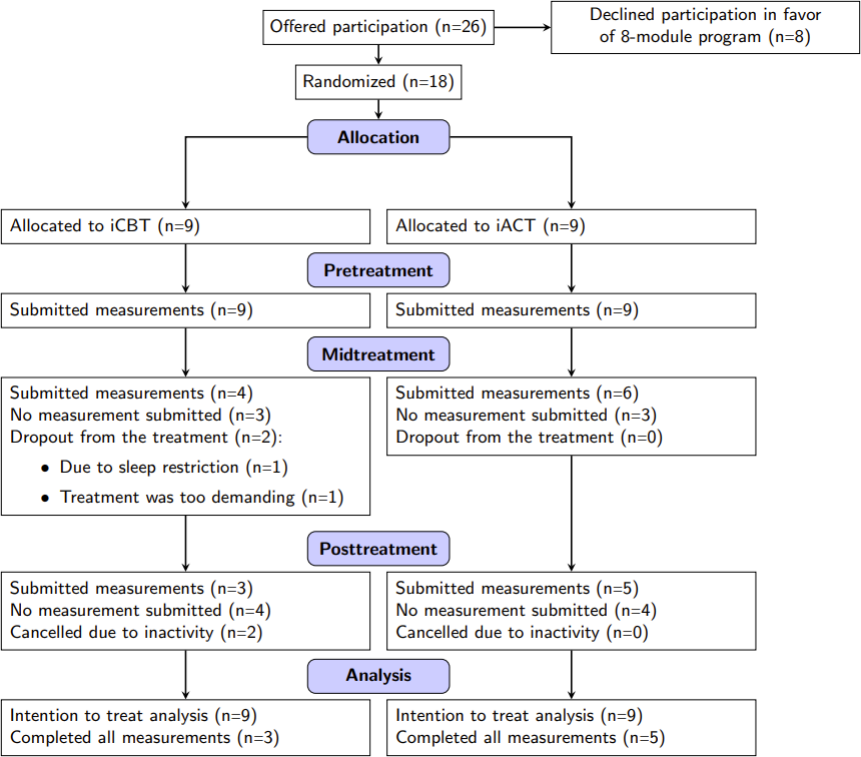
CONSORT (Consolidated Standards of Reporting Trials) flow diagram of participant progress through the recruitment, randomization, and follow-up phases of the internet-delivered cognitive behavioral therapy (iCBT) versus internet-delivered acceptance and commitment therapy (iACT) feasibility trial.

This pilot randomized trial evaluated the feasibility of iCBT and iACT for insomnia, focusing on treatment adherence, the functionality of the digital delivery system, and the practical application of the assessment battery. Participant flow through the trial is illustrated in Figure 1. Due to the pilot nature of the study, exploratory analyses were conducted to observe preliminary trends in clinical outcomes, sleep knowledge, and psychological acceptance, providing a basis for future hypothesis testing. No harms, unintended effects, or consequences were observed.

### Feasibility Outcomes

Recruitment was conducted between 2020 and 2024. Despite extended efforts and the involvement of 16 clinics, only 18 participants were randomized (n=9 per group), falling significantly short of the target sample of 90. Consequently, the trial was deemed unfeasible to continue in its current form after 4 years, resulting in an underpowered sample for inferential testing. No significant secular events, such as technical system outages or major external disruptions, occurred during the study period.

Regarding participant screening, while substance abuse was an exclusion criterion assessed via the MINI interview, subsequent baseline measurements using the AUDIT and DUDIT revealed subclinical usage patterns. The iCBT group reported slightly higher alcohol use than the iACT group (AUDIT mean 4.13, SD 2.70 vs mean 2.11, SD 2.09), respectively. Furthermore, 2 participants in the iCBT group reported low-level drug use (DUDIT scores of 2 and 7). Following clinical review, these participants were retained in the study as their scores did not meet the threshold for dependency, and they fulfilled all other inclusion criteria. The discrepancy between the initial screening and subsequent self-report measures highlights a potential challenge in the screening protocol for future trials. Adherence rates varied between the 2 interventions (Table 3).

**Table 3.** Number of modules initiated by participants in the internet-delivered cognitive behavioral therapy (iCBT) and internet-delivered acceptance and commitment therapy (iACT) groups (n=18)^a^.

Modules	iCBT (n=9), n (%)	iACT (n=9), n (%)
0 (never started)	1 (11)	0 (0)
1	3 (33)	0 (0)
2	0 (0)	1 (11)
3	1 (11)	1 (11)
4	0 (0)	2 (22)
5	4 (44)	5 (55)

a Adherence was defined as the number of modules initiated.

Early dropout was more prevalent in the iCBT group, with 44% (4/9) of the participants discontinuing after the first module or earlier, compared to 0% (0/9) in the iACT group. Specific reasons for early dropout were identified for 2 participants in the iCBT group: one reported that the program was too demanding, and another cited negative prior experiences with sleep restriction. The remaining noncompleters were inactive without providing specific reasons for withdrawal. Overall adherence patterns are further summarized in Figure S1 in Multimedia Appendix 3.

Analysis of CEQ scores revealed no significant main effects for time or group across the treatment period (*P*>.05). Descriptively, however, mean scores for both credibility and expectancy remained high for both groups (Table S1 in Multimedia Appendix 3), suggesting that the treatment rationale was well accepted from the outset and throughout the intervention. Statistical robustness was limited by the small sample size and the restricted number of measurement points. Nonparametric Friedman tests confirmed no significant within-group changes for either the iCBT group (*χ*^2^_1_=0.00; *P*>.99) or the iACT group (*χ*^2^_1_=1.80; *P*=.18). Detailed statistical outputs for the CEQ are provided in Multimedia Appendix 3.

Participant attitudes toward the interventions at pretreatment are detailed in Multimedia Appendix 4. Descriptive data indicated that both groups entered the study with similar baseline profiles. The iCBT group reported slightly higher levels of prior reading regarding their sleep problems (mean 5.44, SD 2.56) compared to the iACT group (mean 4.78, SD 2.68). Conversely, the iACT group rated their initial sleep knowledge marginally higher (mean 4.67, SD 2.00) than the iCBT group (mean 4.33, SD 1.73). Posttreatment, participants in both groups reported increased sleep knowledge (iCBT: *χ*^2^_2_=7.14, *P*=.03; iACT: *χ*^2^_2_=10.89, *P*=.004), with the iCBT group rating the practical usefulness of this knowledge particularly favorably.

Feedback on specific treatment components reflected the distinct nature of each intervention. Participants in the iCBT group highlighted sleep restriction as a core, albeit polarizing, component; while some found it effective for structure, it led to one participant’s withdrawal. In the iACT group, participants emphasized the absence of rigid sleep control and the use of mindfulness exercises as positive aspects of their experience.

Regarding the treatment format, a majority in both groups preferred the internet-delivered blended approach over traditional self-help books, citing the combination of digital modules and psychologist contact as a key strength. While the shortened 5-module format was generally perceived as manageable, technical and engagement barriers were noted (n=2). These included the treatment being perceived as too time-consuming by one participant and a report of an excessive number of short videos by another. Finally, the quality of the sleep journal data was found to be insufficient for inclusion in the formal analysis, suggesting a need for simplified tracking tools in future iterations.

### Process Outcomes

Descriptive analysis of the sleep knowledge test revealed a restricted range of scores and substantial attrition over the course of the study. At pretreatment (n=18), total scores were relatively high in both iCBT (mean 18.67, SD 2.92) and iACT (mean 22.00, SD 2.78) groups.

Despite the small sample size, nonparametric Friedman tests were conducted to explore within-group changes. The results indicated a significant increase in sleep knowledge for both iCBT (*χ*^2^_2_=7.14, *P*=.03) and iACT groups (*χ*^2^_2_=10.89, *P*=.004). However, the substantial data attrition at the final measurement point requires cautious interpretation of these group trends. These observations underscore the feasibility challenges regarding long-term data completeness for the sleep knowledge test within this pilot context. Detailed outputs are provided in Multimedia Appendix 3.

The SAAQ results demonstrated inconsistent trends across statistical models. While the split-plot ANOVA using LOCF indicated significant within-group effects for the iCBT (*F*_2,16_=3.81, *P*=.045), no such effect was observed for the iACT group (*F*_2,16_=0.65, *P*=.53). These findings were not replicated in the CCA (*P*>.05) or supported by nonparametric Friedman tests (iCBT: *χ*^2^_2_=4.22, *P*=.12 and iACT: *χ*^2^_2_=0.61, *P*=.74). Additionally, Kruskal-Wallis tests revealed no significant between-group differences at any measurement point (all *P*>.05). Overall, while some gains in acceptance were descriptively observed, the effects were not statistically robust, and the groups remained comparable at posttreatment. Detailed outputs are provided in Multimedia Appendix 3.

The SPAQ results indicated nonsignificant trends across all analysis methods. When using LOCF (n=18), the main effect was not significant for either the iCBT group (*F*_2,16_=2.32, *P*=.17) or the iACT group (*F*_2,16_=1.19, *P*=.53). Similarly, under CCA, no significant main or within-group effects were observed for either group (*P*>.05). These findings were not supported by the nonparametric Friedman test (iCBT; *χ*^2^_2_=1.71, *P*=.42 and iACT: *χ*^2^_2_=.061, *P*=.74). However, under CCA, no significant main or within-group effects were observed for either group (*P*>.05). While between-group differences remained nonsignificant at all time points according to the Kruskal-Wallis tests (all *P*>.05), posttreatment values for the iCBT group approached significance (*P*=.06). Detailed statistical outputs for the SPAQ are provided in Multimedia Appendix 3.

### Primary Outcomes

Mean scores and preliminary effect sizes for insomnia severity are presented in Table 4.

Both groups showed descriptive improvements in ISI scores from pretreatment to posttreatment. For the iACT group, these within-group improvements were statistically significant under both the LOCF model (*F*_2,16_=5.27, *P*=.004) and the Friedman test (χ^2^_2_=9.52, *P*=.01). In contrast, the iCBT group did not reach statistical significance (*F*_2,16_=2.76, *P*=.097; Friedman test: χ^2^_2_=4.67, *P*=.10). However, these within-group comparisons should be interpreted with caution due to the small sample size and the higher attrition rate observed in the iCBT group, which limited the statistical power to detect significant changes in that arm.

Analysis using CCA also indicated a nonsignificant effect for both the iACT group (*F*_2,8_=2.67, *P*=.13) and the iCBT group (*F*_2,6_=0.57, *P*=.87). Despite these within-group trends, Kruskal-Wallis tests revealed no significant between-group differences at any measurement point (all *P*>.05). These findings suggest that while iACT showed a more consistent preliminary signal of improvement, the study remained underpowered to detect significant differences between the 2 interventions. Detailed statistical outputs are provided in Multimedia Appendix 3.

**Table 4.** Descriptive data and preliminary effect of internet-delivered cognitive behavioral therapy (iCBT) versus internet-delivered acceptance and commitment therapy (iACT) on insomnia severity (n=18) using the last observation carried forward (LOCF).

Measurement	iCBT (n=9), mean (SD)	iACT (n=9), mean (SD)	Statistics^a^
Pretreatment	19.11 (3.44)	19.44 (4.69)	*P*=.87, *d*=0.08 (−0.85 to 1.00)
Midtreatment	16.33 (3.32)	16.89 (3.10)	*P*=.72, *d*=0.17 (−0.76 to 1.01)
Posttreatment	15.55 (5.88)	13.89 (4.62)	*P*=.51, *d*=−0.28 (−1.21 to 0.66)

a Statistics (*P* value, *d* and 95% CI) refer to between-group comparisons at each time point; all measures used the full intention-to-treat sample (n=18).

### Secondary Outcomes

Secondary outcomes generally lacked statistical significance across both LOCF and CCA analyses. While mean scores for anxiety (GAD-7) and functional impairment (WHODAS) showed slight downward trends in both iCBT and iACT groups, these changes did not reach significance in parametric (all *P*>.05) or nonparametric testing (all *P*>.20). Specifically, within-group changes for GAD-7 scores remained nonsignificant for both iCBT (*χ*^2^_2_=1.62, *P*=.44) and iACT (*χ*^2^_2_=0.67, *P*=.72). Similarly, no significant effects were observed for WHODAS (iCBT: *P*=.61; iACT: *P*=.45). Overall, no clear clinical signal for comorbid symptoms or functional improvement was detected within this pilot sample, likely due to limited statistical power. Detailed statistical tables and raw outputs are provided in Multimedia Appendix 3.

## Discussion

### Principal Findings: Efficacy and Clinical Trends

This pilot RCT provides preliminary insights into the clinical trends of iCBT and iACT for insomnia within a primary care context. Regarding sleep outcomes, only the iACT group reached statistical significance in within-group ISI improvements. However, this likely reflects differences in statistical power rather than true clinical superiority, as the iCBT group experienced higher attrition. Despite these trends, the study did not exhibit a significant interaction effect, and the small sample size (n=18) precludes any firm conclusions regarding the comparative efficacy of the 2 modalities.

Secondary outcomes, including depression (PHQ-9), anxiety (GAD-7), and general functioning (WHODAS), did not reach statistical significance in any primary or sensitivity analyses. While the interventions may have a positive directional influence on comorbid symptoms, the lack of a clear clinical signal suggests that these results should be viewed as exploratory. Participant perceptions of treatment credibility and expectancy (CEQ) remained relatively stable, with no distinct differences observed between the groups [25,26]. In accordance with CONSORT-eHEALTH guidelines, no subgroup analyses (eg, based on age or baseline severity) were performed due to the limited sample size, which would have rendered such comparisons statistically unstable.

### Feasibility: Recruitment, Adherence, and Format

The primary barrier to feasibility was the low recruitment rate. Despite involving 16 clinics over 4 years, the yield was insufficient, likely due to competition with established treatment pathways, such as traditional “sleep schools.” Patients were likely triaged to these familiar interventions, leaving internet-delivered therapy without a clear position in the natural patient flow.

Furthermore, the attempt to enhance accessibility by shortening the intervention from 8 to 5 modules [15] appeared to backfire for a subset of potential participants; 8 individuals explicitly declined participation because they preferred a more extensive program. This suggests that while some find digital interventions demanding, others may perceive a shorter format as insufficient. Despite the shortened format, approximately half of the participants dropped out before the final module. High attrition and assessment burden likely impacted engagement, with difficulties implementing techniques like sleep restriction potentially contributing to nonadherence in iCBT [8]. As suggested by Linares et al [13], iACT may serve as a valuable alternative for patients who find the behavioral requirements of iCBT too demanding.

### The Role of Process Variables and Knowledge

The 30-item sleep knowledge test was developed as a “proxy measure” for engagement to evaluate whether participants internalized the treatment rationale. While preliminary piloting with health care staff [27] ensured item intelligibility, the current trial revealed restricted variability and potential “guessing bias.” The lack of significant within-group changes in knowledge scores, particularly in the iCBT arm, underscores the need for formal psychometric validation to increase sensitivity to treatment-specific gains.

Similarly, few statistically significant changes were observed in psychological flexibility (SAAQ) or sleep-related acceptance (SPAQ). While some nominal effects emerged for the SAAQ and SPAQ when using the LOCF method, these did not hold under CCA or nonparametric testing. Given the inherent biases of LOCF in small samples [38], these observations must be interpreted with caution. It remains unclear whether the absence of consistent significance reflects a true lack of effect or a failure to detect meaningful shifts due to the underpowered nature of the pilot study.

### Strengths, Limitations, and Future Directions

A primary strength of this study is its high ecological validity, as participants were recruited from the regular patient flow within a national eHealth infrastructure (the SOB platform). However, the small sample size and high proportion of missing data limit the generalizability of the findings. The recruitment period overlapped with the COVID-19 pandemic, which may have impacted adherence and sleep patterns, though these factors were not explicitly assessed. Furthermore, the comprehensive assessment battery, totaling 30 knowledge items alongside multiple clinical scales, likely imposed a high respondent burden, contributing to the observed attrition.

Future research should prioritize positioning digital interventions as a first-line stepped-care option [41], ensuring they are integrated early into the primary care clinical pathway. To improve the yield of randomized participants, studies should use more active recruitment strategies, such as follow-up phone calls [42] and automated reminders. Additionally, using a planned missing data design [43] could reduce respondent burden and improve data completeness. Finally, refining the sleep knowledge test to include objective behavioral metrics, such as data from wearable sleep trackers, would allow for a more robust evaluation of how theoretical knowledge translates into behavioral change.

## Supplementary material

10.2196/81285 Multimedia Appendix 1 Informed consent form and participant information sheet (Swedish).

10.2196/81285 Multimedia Appendix 2 Sleep knowledge test (English translation).

10.2196/81285 Multimedia Appendix 3 Supplementary statistical results.

10.2196/81285 Multimedia Appendix 4 Participant attitudes toward treatment at pretreatment and posttreatment.

10.2196/81285 Checklist 1 CONSORT-eHEALTH checklist (V 1.6.1).

## Abbreviations

ACT: acceptance and commitment therapy
AUDIT: Alcohol Use Disorders Identification Test
CCA: Complete Case Analysis
CEQ: Credibility/Expectancy Questionnaire
CHERRIES: Checklist for Reporting Results of Internet E-Surveys
CONSORT: Consolidated Standards of Reporting Trials
*DSM-5*: *Diagnostic and Statistical Manual of Mental Disorders, Fifth Edition*
DUDIT: Drug Use Disorders Identification Test
GAD-7: 7-item Generalized Anxiety Disorder
iACT: Internet-delivered Acceptance and Commitment Therapy
iCBT: internet-delivered cognitive behavioral therapy
*ICD-10*: *International Classification of Diseases, 10th Revision*
ISI: insomnia severity index
LOCF: last observation carried forward
MINI: Mini International Neuropsychiatric Interview
PHQ-9: 9-item Patient Health Questionnaire
RCT: randomized controlled trial
SAAQ: Swedish Acceptance and Action Questionnaire
SOB: Stöd och behandling
SPAQ: Sleep Problem Acceptance Questionnaire
VGR: Västra Götaland Region
WHODAS: WHO Disability Assessment Schedule
