# Supplementary material for: Conceptualizing Acceptance and Knowledge as Process Variables in Internet-Delivered and Therapist-Supported Cognitive Behavioral Therapy and Acceptance and Commitment Therapy in Primary Care for Insomnia: Pilot Feasibility and Process-Oriented Randomized Controlled Trial
Source: JMIR Form Res. 2026 May 21;10:e81285. doi: 10.2196/81285 (PMC13193663; doi:10.2196/81285)
Supplement: Multimedia Appendix 1 [file formative-v10-e81285-s001.pdf]

VÄSTRA GÖTALANDSREGIONEN

Information till forskningspersonerna – patienter primärvård

Du har meddelat intresse av att delta i ett forskningsprojekt. I det här dokumentet får du information om projektet och om vad det innebär att delta.

Vad är det för projekt och varför vill ni att jag ska delta?

Effekten av *internetförmedlad kognitiv beteendeterapi* (iKBT), för vuxna med sömnbesvär är väldokumenterad. I flera internationella studier har man sett att iKBT dessutom fungerar bra mot bland annat panikångest, depression, huvudvärk, tinnitus och posttraumatiskt stressyndrom (PTSD). Primärvården har även tidigare genomfört projekt med iKBT för vuxna med psykisk ohälsa med goda resultat. Trots goda resultat av iKBT är avhopp från behandlingen vanligt. För att minska avhopp och öka genomförande av behandling kan det vara nödvändigt med ett utökat stöd för att handskas med sina symptom under tiden man genomgår behandlingen.

På många av Närhälsans vårdcentraler erbjuds en internetbaserad behandling mot sömnbesvär. Behandlingen innehåller en kombination av *Kognitiv BeteendeTerapi* (KBT) och *Acceptance and Commitment Therapy* (ACT). Den här studien syftar till att undersöka om det går att göra behandlingen mer effektiv genom att dela upp den åtta veckor långa behandlingen till två olika versioner på fem veckor vardera och sedan jämföra dem med varandra.

Studien är en *randomiserad kontrollerad studie* (RCT) vilket innebär att du som patient kommer att lottas till en av två grupper. Om du lottas till den ena gruppen genomgår du en fem veckor lång behandling i *internetbaserad Kognitiv BeteendeTerapi* (iKBT) mot sömnproblem och om du lottas till den andra gruppen får du en fem veckor lång *internetbaserad Acceptance and Commitment Therapy* (iACT) mot sömnproblem.

Studien vänder sig till dig som är äldre än 18 år, med sömnbesvär, och som söker hjälp inom primärvården.

Forskningshuvudman för projektet är Primärvårdsstyrelsen. Med forskningshuvudman menas den organisation som är ansvarig för studien.

För att kunna vara med i studien krävs att du

- förstår talad och skriven svenska
- har tillgång till dator/surfplatta/smartphone med internetsurf.

Hur går studien till?

Om du deltar i studien kommer du få svara på frågor via mailutskick och i själva behandlingsprogrammet, när du loggar in via 1177. Det är till för att vi skall kunna se vilken effekt behandlingen har. Du kommer också att få fylla i formulär en tid efter behandlingens slut. Detta för att se om behandlingens effekt finns kvar över längre tid.

Möjliga följder och risker med att delta i studien

Den risk som ditt deltagande i studien möjligen kan innebära är att behandlingen inte passar dig. Om du känner att du inte längre vill vara med i studien kan du säga till din behandlare. Din behandling kommer då att fortsätta fast på annat sätt. Ibland kan det kännas jobbigt att gå igenom behandlingar som detta på internet, men på längre sikt kan det göra att du mår bättre.

Att delta i studien kan också innebära att det kan kräva något mer tid av dig att svara på frågor kopplat till din psykiska hälsa som behövs för att kunna utvärdera behandlingens effekt. När du svarar på frågor så som dem som kommer att skickas ut till dig i studien, kan det väcka känslor hos dig som patient. Du kan i så fall kontakta din behandlare.

Vad händer med mina uppgifter?

Projektet kommer att samla in och registrera information om dig.

Dina svar i frågeformulär via 1177 före, under och efter behandlingen kommer efter ditt deltagande att förvaras avkodade vid primärvårdens FoU-centrum Södra Älvsborg, Sven Eriksonsplatsen 4, Borås. Ingen obehörig kommer att kunna se hur just du svarade. När din behandling i studien är klar kommer informationen om dig, alla svar du gett i projektet, att flyttas till låst skåp på Primärvårdens forskningsenhet (FoU-enhet) på Sven Eriksonsplatsen 4 i Borås. Där kommer sedan all information från projektet att bearbetas och användas i forskning.

Det är bara personer som arbetar med just vårt projekt som kan komma åt informationen. Samtliga svar, frågeformulär och testresultat kommer att kodas. Den kodade listan liksom svaren kommer att förvaras inlåsta först på mottagningen och sedan på FoU-enheten, Sven Eriksonplatsen 4 i Borås. Allt material kommer att kodas. Kodningslistan kommer att förvaras separat, i låst skåp, på FoU-enheten. All redovisning av intervjuer, sammanställning och publikation av resultat kommer att ske så att uppgiftslämnare inte kan identifieras.

Dina svar och dina resultat kommer att behandlas så att inte obehöriga kan ta del av dem.

Primärvårdsstyrelsen är personuppgiftsansvarig. Enligt EU:s dataskyddsförordning har du rätt att kostnadsfritt få ta del av de uppgifter om dig som hanteras i studien, och vid behov få eventuella fel rättade. Du kan också begära att uppgifter om dig raderas samt att behandlingen av dina personuppgifter begränsas.

Om du vill ta del av uppgifterna ska du kontakta studieansvarig forskare Sandra Weineland, sandra.weineland@vgregion.se, primärvårdens FoU centrum, FoU-enheten, Sven Eriksonplatsen 4 i Borås. Har du frågor eller synpunkter kring hanteringen av dina

personuppgifter kan du även kontakta Närhälsans dataskyddsombud som du når på: narhalsan.dataskyddsombud@vgregion.se. Om du är missnöjd med hur dina personuppgifter behandlas har du rätt att ge in klagomål till Datainspektionen, som är tillsynsmyndighet.

Hur får jag information om resultatet av studien?

Resultaten från studien kommer att publiceras i vetenskapliga tidskrifter. Alla resultat sammanställs och redovisas så att det inte framgår vem som svarat vad. Om du vill ha en sammanställning av studiens resultat, eller en sammanställning av dina egna svar, så kan du kontakta oss om det. Kontaktinformation till oss finns längst ned detta dokument. Vi kommer inte att automatiskt skicka några uppgifter till dig.

Ersättning

Du får ingen ersättning för deltagande i studien.

Deltagandet är frivilligt

Ditt deltagande är frivilligt och du kan när som helst välja att avbryta deltagandet. Om du väljer att inte delta eller vill avbryta ditt deltagande behöver du inte uppge varför, och det kommer inte heller att påverka din framtida vård eller behandling eller kontakt med projektet.

Om du vill avbryta ditt deltagande ska du kontakta den ansvariga för studien (se nedan).

Ansvariga för studien

Studien utförs inom plattformen Psykisk hälsa och ohälsa vid Närhälsan i Västra Götalandsregionen och leds av docent Sandra Weineland, primärvårdens FoU-centrum Södra Älvsborg, Sankt Eriksplan 4, Borås.

Forskningshuvudman och personuppgiftsansvarig är Primärvårdsstyrelsen.

Huvudansvarig forskare är docent Sandra Weineland, primärvårdens FoU-centrum Södra Älvsborg, Sankt Eriksplan 4, Borås, telefonnummer: 072-4541252 och e-post: sandra.weineland@vgregion.se.

Kontakt och mer information:
Anna Larsson
Leg psykolog
anna.caroline.larsson@vgregion.se

Sandra Weineland
Leg psykolog
Docent i psykologi
Telefonnummer: 072-4541252
sandra.weineland@vgregion.se

Samtycke till att delta i studien

Jag har fått muntlig och skriftlig informationen om studien *Jämförelse mellan iKBT och iACT vid sömnbesvär: acceptans och kunskapsinhämtning som verksamma mekanismer. En randomiserad kontrollerad studie* och har haft möjlighet att ställa frågor.

Genom att markera ”Jag godkänner informationen och vill delta i forskningsprojektet. ” i den digitala versionen av den här informationen samtycker jag till att delta i studien. Jag samtycker i och med det också till att uppgifter om mig behandlas på det sätt som beskrivs i den här informationen samt att data/uppgifter från journal får inhämtas.
