# Supplementary material for: Conceptualizing Acceptance and Knowledge as Process Variables in Internet-Delivered and Therapist-Supported Cognitive Behavioral Therapy and Acceptance and Commitment Therapy in Primary Care for Insomnia: Pilot Feasibility and Process-Oriented Randomized Controlled Trial
Source: JMIR Form Res. 2026 May 21;10:e81285. doi: 10.2196/81285 (PMC13193663; doi:10.2196/81285)
Supplement: Multimedia Appendix 2 [file formative-v10-e81285-s002.pdf]

Knowledge About Sleep

You will now answer questions about sleep. Some questions are easier, others more difficult. The important thing is that you answer as best you can — without thinking for too long. You are not expected to know all the answers. In some cases, more than one answer option may seem correct; in that case, choose the one that feels most correct. No aids may be used.

Sleep

1.

a) What has the greatest impact on the circadian rhythm?

1. **The alternation between light and darkness.**
2. Our eating and sleeping habits.
3. Our age.

b) How confident are you in your answer?
1 2 3 4 5
1 = Very uncertain   5 = Very certain

2.

a) Is there a difference in body temperature when we are asleep compared to when we are awake?

1. **Body temperature is lower when we sleep.**
2. Body temperature remains at the same level regardless of whether we are asleep or awake.
3. Body temperature is higher when we sleep.

b) How confident are you in your answer?
1 2 3 4 5
1 = Very uncertain   5 = Very certain

3.

a) What is measured during EEG recordings?

1. **Electrical activity**
2. Muscle twitches
3. Hormones

b) How confident are you in your answer?
1 2 3 4 5
1 = Very uncertain 5 = Very certain

4.

a) As we age, we need less sleep — but which type of sleep primarily decreases?

1. Light sleep
2. **Deep sleep**
3. Dream sleep

b) How confident are you in your answer?
1 2 3 4 5
1 = Very uncertain 5 = Very certain

5.

a) Can the body recover lost sleep?

1. No.
2. **Yes, by sleeping more deeply.**
3. Yes, by sleeping longer.

b) How confident are you in your answer?
1 2 3 4 5
1 = Very uncertain 5 = Very certain

6.

a) Is there often a difference between how much we think we sleep and how much we actually sleep?

1. Yes, most people sleep less than they think.
2. No, most people have an accurate perception of how much they sleep.
3. **Yes, most people sleep more than they think.**

b) How confident are you in your answer?
1 2 3 4 5
1 = Very uncertain   5 = Very certain

7.

a) What has the greatest impact on how sleepy we feel when we wake up?

1. How long we have slept.
2. Sleep quality.
3. **The sleep stage from which we woke up.**

b) How confident are you in your answer?
1 2 3 4 5
1 = Very uncertain   5 = Very certain

8.

a) Which factors have the greatest impact on how we sleep?

1. **How long we have been awake, the circadian rhythm, and stress level.**
2. The sleep environment in terms of light, sound, and temperature.
3. Whether one has consumed coffee and/or alcohol during the day.

b) How confident are you in your answer?
1 2 3 4 5
1 = Very uncertain   5 = Very certain

9.

a) How common is it to wake briefly during sleep?

1. We usually sleep without interruption.
2. It only happens to certain people, and they usually remember it afterward.
3. **Everyone wakes up from time to time during sleep but usually does not remember it.**

b) How confident are you in your answer?
1 2 3 4 5
1 = Very uncertain   5 = Very certain

10.

a) How is the circadian rhythm related to the sleep hormone melatonin?

1. **Exposure to daylight reduces melatonin production.**
2. Exposure to daylight increases melatonin production.
3. The body produces more melatonin during times of day when there is usually a relatively high amount of sunlight.

b) How confident are you in your answer?
1 2 3 4 5
1 = Very uncertain   5 = Very certain

ICBT

11.

a) What does the treatment approach CBT stand for?

1. **Cognitive Behavioral Therapy**
2. Cognition-Based Therapy
3. Knowledge and Needs Therapy

b) How confident are you in your answer?
1 2 3 4 5
1 = Very uncertain   5 = Very certain

12.

a) How should one think about bedtimes if experiencing sleep problems?

1. Go to bed early to increase the chance of getting as much sleep as possible.
2. **Maintain regular bedtimes regardless of how one slept the night before.**
3. Sleep in when possible, especially after a poor night’s sleep or when feeling extra sleepy.

b) How confident are you in your answer?
1 2 3 4 5
1 = Very uncertain   5 = Very certain

13.

a) What can be done during the day to improve nighttime sleep?

1. **Exercise, eat regularly, and reduce stress.**
2. Rest frequently during the day and take naps when tired.
3. Drink coffee to stay active during the day.

b) How confident are you in your answer?
1 2 3 4 5
1 = Very uncertain   5 = Very certain

14.

a) What can be done to increase sleep efficiency?

1. **Spend less time in bed.**
2. Spend the same amount of time in bed as before.
3. Spend more time in bed.

b) How confident are you in your answer?
1 2 3 4 5
1 = Very uncertain   5 = Very certain

15.

a) What should one do if feeling sleepy during the day?

1. Reduce activities to avoid overexertion.
2. Take a nap.
3. **Continue as usual.**

b) How confident are you in your answer?
1 2 3 4 5
1 = Very uncertain   5 = Very certain

16.

a) How should meals be distributed to optimally promote sleep?

1. Eat the last meal as early as possible to avoid digestion disturbing sleep.
2. **Distribute nutrient intake across several smaller meals to keep blood sugar stable.**
3. Eat the last meal right before bedtime to reduce the risk of waking due to hunger.

b) How confident are you in your answer?
1 2 3 4 5
1 = Very uncertain   5 = Very certain

17.

a) What is a power nap?

1. A nap that includes deep sleep.
2. **A very short nap.**
3. A nap taken in preparation for physical or mental exertion.

b) How confident are you in your answer?
1 2 3 4 5
1 = Very uncertain   5 = Very certain

18.

a) What does sleep restriction mean?

1. **Aiming to sleep according to one's average actual sleep time, then gradually extending it.**
2. Aiming to have as short a sleep time as one can tolerate, then gradually extending it.
3. Getting out of bed for a while if unable to fall asleep.

b) How confident are you in your answer?
1 2 3 4 5
1 = Very uncertain   5 = Very certain

19.

a) What does sleep compression mean?

1. Aiming to sleep according to one's average actual sleep time, then gradually extending it.
2. **Aiming to have as short a sleep time as one can tolerate, then gradually extending it.**
3. Getting out of bed for a while if unable to fall asleep.

b) How confident are you in your answer?
1 2 3 4 5
1 = Very uncertain 5 = Very certain

20.

a) What does stimulus control mean?

1. Aiming to sleep according to one's average actual sleep time, then gradually extending it.
2. Aiming to have as short a sleep time as one can tolerate, then gradually extending it.
3. **Getting out of bed for a while if unable to fall asleep.**

b) How confident are you in your answer?
1 2 3 4 5
1 = Very uncertain 5 = Very certain

ACT

21.

a) What does the treatment approach ACT stand for?

1. **Acceptance and Commitment Therapy**
2. Applied Cognitive Theory
3. Altruistic Compassion Treatment

b) How confident are you in your answer?
1 2 3 4 5
1 = Very uncertain 5 = Very certain

22.

a) Why is it important to be able to accept negative thoughts and feelings related to sleep?

1. **When you accept the thoughts, they become less intrusive, which in turn benefits sleep.**
2. To more easily get rid of negative thoughts and feelings so you can focus on sleeping instead.
3. So that positive thoughts become clearer and easier to focus on.

b) How confident are you in your answer?
1 2 3 4 5
1 = Very uncertain   5 = Very certain

23.

a) Is it helpful for sleep to mentally try hard to fall asleep?

1. Yes, it tires the brain, making it easier to fall asleep.
2. No, it is better to take it as easy as possible during the day.
3. **No, it tends to increase worry and makes it harder to fall asleep.**

b) How confident are you in your answer?
1 2 3 4 5
1 = Very uncertain   5 = Very certain

24.

a) In what way can focusing on other areas of life be helpful for sleep?

1. **It improves overall well-being, which can reduce stress and worry and thereby positively affect sleep.**
2. Because one should first solve daytime problems in order to then be able to focus on sleep.
3. To ensure you have something better to do instead of lying awake.

b) How confident are you in your answer?
1 2 3 4 5
1 = Very uncertain   5 = Very certain

25.

a) What does mindfulness mean?

1. Trying to think as little as possible.
2. Relaxing.
3. **Being aware of one's thoughts and feelings.**

b) How confident are you in your answer?
1 2 3 4 5
1 = Very uncertain   5 = Very certain

26.

a) What does it mean to use the visualization method to manage disturbing thoughts?

1. Observing thoughts and feelings without acting on them.
2. **Imagining an inner image in which you are calm and relaxed in order to train attention.**
3. Scheduling a specific time to worry about things.

b) How confident are you in your answer?
1 2 3 4 5
1 = Very uncertain   5 = Very certain

27.

a) What does the rule of thumb OAL mean?

1. **Observe – Accept – Step back**
2. **O**vercome – **A**ct – **S**ettle down
3. **O**ptimize – **A**pply – **S**leep

b) How confident are you in your answer?
1 2 3 4 5
1 = Very uncertain   5 = Very certain

28.

a) What are mirror neurons and how do they affect us?

1. They are activated when we see our reflection, allowing us to ignore that it is reversed.
2. **They help us perceive others' emotional expressions and unconsciously mimic facial expressions, which in turn makes us feel similarly.**
3. They are activated when we see ourselves and influence how we perceive ourselves.

b) How confident are you in your answer?
1 2 3 4 5
1 = Very uncertain 5 = Very certain

29.

a) In what way can mindfulness be helpful?

1. **By becoming more aware of thoughts, feelings, and problems so that one can relate to them more effectively.**
2. By gaining better control over thoughts and feelings.
3. By becoming better at distracting oneself from unwanted thoughts or feelings.

b) How confident are you in your answer?
1 2 3 4 5
1 = Very uncertain 5 = Very certain

30.

a) How can one relate to threatening thoughts and feelings?

1. Regard them as truths and adapt one's life accordingly.
2. Ignore them and focus instead on what is positive in life.
3. **Actively choose how to act and allow the thoughts and feelings to be present.**

b) How confident are you in your answer?
1 2 3 4 5
1 = Very uncertain 5 = Very certain

Description: A 30-item developmental instrument assessing knowledge of sleep disorders, CBT-I, and ACT-I principles. Participants were tested at pre-, mid-, and posttreatment. Each question includes one correct and two incorrect options.
