# Supplementary material for: Conceptualizing Acceptance and Knowledge as Process Variables in Internet-Delivered and Therapist-Supported Cognitive Behavioral Therapy and Acceptance and Commitment Therapy in Primary Care for Insomnia: Pilot Feasibility and Process-Oriented Randomized Controlled Trial
Source: JMIR Form Res. 2026 May 21;10:e81285. doi: 10.2196/81285 (PMC13193663; doi:10.2196/81285)
Supplement: Multimedia Appendix 3 [file formative-v10-e81285-s003.docx]

Supplementary statistical results

*1. Feasibility Outcomes*

*Treatment adherence*

This figure illustrates the proportion of participants in the iCBT (green line) and iACT (blue line) groups who remained active in the trial across the four treatment modules.

**Figure S1.** Kaplan-Meier adherence curve


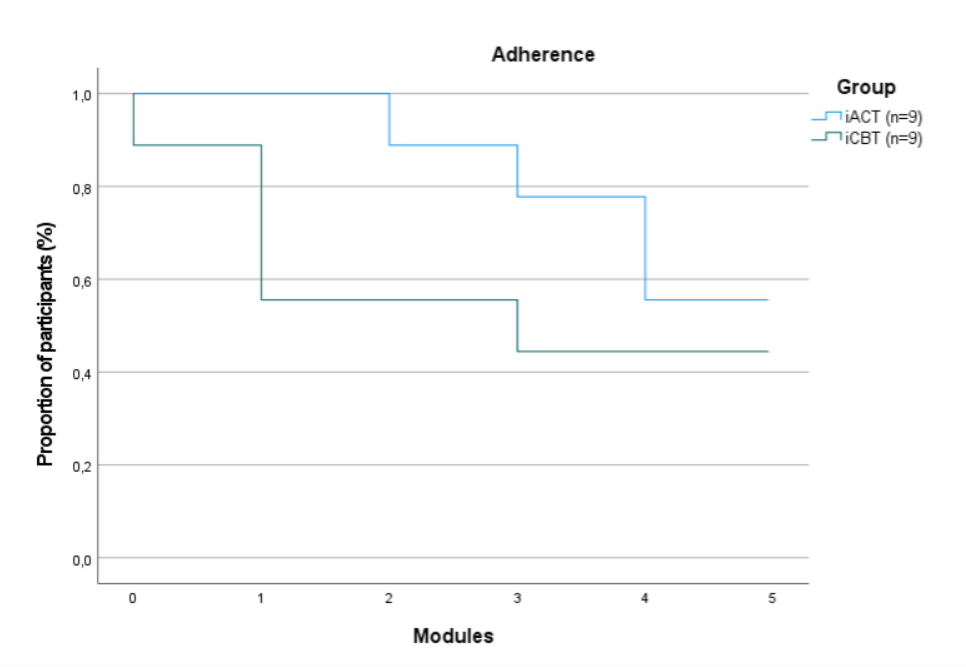
**Note:** Steps represent participant dropouts at each module transition.

Abbreviations: iACT: internet-delivered Acceptance and Commitment Therapy; iCBT: internet-delivered Cognitive Behavioral Therapy.

*CEQ*

CEQ was analyzed to assess treatment rationale acceptance (see Table S1).

**Table S1.** Results of *CEQ* using observed case analysis.

|  | iCBT Mean (SD) | iACT Mean (SD) |
| --- | --- | --- |
| Pretreatment | n=9 | n=9 |
|  | 35.89 (7.93) | 37.00 (11.30) |
| Midtreatment | n=4 | n=6 |
|  | 37.25 (3.20) | 34.83 (10.83) |

**Note:** Abbreviations: CEQ: Credibility/Expectancy Questionnaire; iACT: internet-delivered Acceptance and Commitment Therapy; iCBT: internet-delivered Cognitive Behavioral Therapy; *n*: number of participants with available data at that time point; SD: standard deviation.

In the LOCF analysis (N=18), the main effect of time remained non-significant for both the iCBT group (*F*_1,8_=0.00,*P*=1.00) and the iACT group (*F*_1,8_=1.78,*P*=.22). Friedman tests using LOCF further supported this lack of significant change for iCBT (*χ* ^2^_1_=0.00,*P*=1.00) and iACT (*χ* ^2^_1_=1.80,*P*=.18). These findings suggest that treatment credibility and expectancy remained stable and high throughout the mid-treatment phase for both interventions.

A split-plot ANOVA using CCA (n=4 for iCBT; n=6 for iACT) showed no significant main effect of time for the iCBT group (*F*_1,3_=1.08,*P*=.37) or the iACT group (*F*_1,5_=1.19,*P*= .33). Non-parametric Friedman tests confirmed these results, indicating no significant within-group changes for iCBT (*χ*^2^_1_=0.55,*P*=.76) or iACT ( *χ* ^2^_1_=0.40,*P*=.82).

*2. Process Outcomes*

**Table S2.** Results of the Sleep knowledge test across treatment phases

using observed case analysis.

|  | **iCBT Mean (SD)** | **iACT Mean (SD)** |
| --- | --- | --- |
| Pretreatment | n=9 | n=9 |
| 1-10: Sleep and sleep disorders | 5.22 (2.17) | 6.78 (1.48) |
| 11-20: CBT for insomnia | 6.22 (1.09) | 7.00 (1.87) |
| 21-30: ACT for insomnia | 7.33 (1.87) | 7.78 (0.97) |
| Total | 18.67 (2.92) | 22.00 (2.78) |
|  |  |  |
| Midreatment | n=4 | n=6 |
| 1-10: Sleep and sleep disorders | 7.25 (1.71) | 6.67 (2.07) |
| 11-20: CBT for insomnia | 8.00 (1.41) | 7.33 (1.51) |
| 21-30: ACT for insomnia | 7.25 (1.50) | 7.17 (1.72) |
| Total | 22.75 (1.26) | 21.33 (4.50) |
|  |  |  |
| Posttreatment | n=3 | n=4 |
| 1-10: Sleep and sleep disorders | 7.67 (0.58) | 7.50 (0.58) |
| 11-20: CBT for insomnia | 7.00 (1.00) | 4.75 (0.96) |
| 21-30: ACT for insomnia | 8.67 (2.31) | 8.50 (0.58) |
| Total | 23.33 (2.08) | 20.50 (2.08) |

**Note:** This table uses an observed case analysis approach; *n*-values represent the number of participants who submitted measurements at each specific time point. Abbreviations: iACT: internet-delivered Acceptance and Commitment Therapy; iCBT: internet-delivered Cognitive Behavioral Therapy.

*SAAQ*

In the LOCF analysis (N=18), the split-plot ANOVA showed a statistically significant within-group effect for the iCBT-group (*F*_2,16_=3.81,*P*=.045) but not the iACT group (*F*_2,16_=0.65,*P*=.53). Friedman tests using LOCF indicated no significant within-group differences for the iCBT (*χ* ^2^_2_=4.22,*P*=.12) or iACT groups (*χ* ^2^_2_=0.61,*P*=.74). Similarly, Kruskal-Wallis tests using LOCF found no significant between-group differences at any time point (*P* range: .59 to .93).

To evaluate changes in psychological acceptance, a split-plot ANOVA and non-parametric tests were conducted using CCA (see Table S2). The main effect of time on SAAQ scores was not significant for either the iCBT group (*F*_2,6_=2.20,*P*=.39) or the iACT group (*F*_2,8_=0.17,*P*=.85). Friedman tests supported these findings, showing no significant within-group changes for iCBT (*χ* ^2^_2_=3.82,*P*=.15) or iACT (*χ* ^2^_2_=1.20,*P*=.55). Kruskal-Wallis tests indicated no statistically significant between-group differences at pretreatment *P*=.88), midtreatment (*P*=.55), or posttreatment (*P*=.55).

*SPAQ*

In the LOCF analysis (N=18), the main effect of time was not significant for the iCBT group (*F*_2,16_=2.32,*P*=.17) or the iACT group (*F*_2,16_=1.19,*P*=.53). Friedman tests using LOCF further supported this, showing no significant within-group change for iCBT (*χ*^2^_2_=1.71,*P*=.42) or iACT (*χ*^2^_2_=0.61,*P*=.74). Kruskal-Wallis tests indicated no significant between-group differences at any time-point (*P range: .*06 to .40).

To evaluate changes in sleep prioritization, a split-plot ANOVA and non-parametric tests were conducted using CCA. The main effect of time on SPAQ scores was not significant for either the iCBT group (*F*_2,6_=18.08,*P*=.065) or the iACT group (*F*_2,3_=1.19,*P*=.65). Non-parametric Friedman tests confirmed these findings, indicating no significant within-group differences for iCBT (*χ*^2^_2_=0.55, *P*=.76) or iACT ( *χ*^2^_2_=0.40,*P*=.82). Kruskal-Wallis tests showed no statistically significant between-group differences at any time point (*P* range: .29 to 1.00).

*3. Primary outcomes*
In the LOCF analysis (N=18), the split-plot ANOVA showed that the main effect of time was not significant for the iCBT group (*F*_2,16_=2.76,*P*=.097; Friedman test: *χ ^2^*_2_=4.67,*P*=.10), but a significant within-group improvement was observed for the iACT group (*F*_2,16_=5.27,*P*=.004; Friedman test: *χ^2^*_2_=9.52,*P*=.01). Despite the within-group improvement in the iACT arm, Kruskal-Wallis tests revealed no statistically significant between-group differences at pretreatment (*P*=.79), mid-treatment (*P*=.76), or post-treatment (*P*=.43).

The CCA yielded consistent results. The main effect of time was non-significant for the iCBT group (*F*_2,6_=1.84,*P*=.24). For the iACT group, the improvement remained descriptively stronger but did not reach significance in the small complete-case sample (*F*_2,8_=2.67,*P*=.14). Friedman tests for the CCA indicated no significant within-group differences for iCBT (*χ ^2^*_2_=4.67,*P*=.10) or iACT ( *χ ^2^*_2_=4.53,*P*=.10). Between-group comparisons using Kruskal-Wallis tests remained non-significant across all time points (*P* range: .29 to .76).

*4. Secondary Outcomes*

The following table presents the descriptive statistics for comorbid symptoms of depression, anxiety, and functional impairment. These measures were included as exploratory endpoints. While some isolated effects were observed in the LOCF models, the lack of consistency across analyses (CCA vs. LOCF) and the small pilot sample size suggest these findings should be interpreted as preliminary and non-robust.

**Table S3.** Descriptive statistics for secondary outcomes (PHQ-9, GAD-7, and WHODAS) using LOCF

| **Measurement** | **iCBT M (SD)** | **iACT M (SD)** | **Statistics** |
| --- | --- | --- | --- |
| Depression (PHQ-9) | (n=9) | (n=9) |  |
| Pretreatment | 14.67 (6.19) | 11.78 (8.04) | *P*=.40, *d*=-.40 [1.33, 0.54] |
| Posttreatment | 12.44 (6.95) | 9.11 (4.57) | *P*=.25, *d*=-.57 [-1.50, 0.39] |
| Anxiety (GAD-7) | (n=9) | (n=9) |  |
| Pretreatment | 8.25 (4.98) | 9.33 (6.61) | *P*=.18, *d*=.71 [0.77, 1.14] |
| Midtreatment | 7.13 (4.32) | 9.33 (5.98) | *P*=.40, *d*=.42 [-0.52, 1.38] |
| Posttreatment | 6.75 (4.17) | 7.78 (5.07) | *P*=.66, *d*=.22 [0.74, 1.17] |
| Function (WHODAS) | (n=8) | (n=9) |  |
| Pretreatment | 20.25 (16.95) | 10.67 (8.40) | *P* =.15, *d*=.73 [-1.71, 0.27] |
| Midtreatment | 16.13 (8.76) | 10.78 (7.87) | *P* =.21, *d*=.65 [-1.61, 0.35] |
| Posttreatment | 17.13 (10.01) | 9.56 (6.86) | *P* =.09, *d*=.89 [-1.88, 0.12] |

**Note:** All clinical outcomes were analyzed using last observation carried forward (LOCF) to maintain the intention-to-treat sample. Preliminary effect sizes (*d*) represent Cohen *d*. Abbreviations: CI: confidence interval; *d*: Cohen *d*; iACT: internet-delivered acceptance and commitment therapy; iCBT: internet-delivered cognitive behavioral therapy; ISI: Insomnia Severity Index; LOCF: last observation carried forward; *M*: mean; *n*: number of participants; *P*: *P* value; *SD*: standard deviation.

Due to missing data at baseline, WHODAS analyses were conducted using complete case analysis (n=8) for iCBT; (n=9) for iACT), while all other measures utilized the full sample (n=9 per group).

*PHQ-9*

In the split-plot ANOVA using LOCF, the main effect was not significant for the iCBT group (*F*_1, 8_=1.61,*MSE*=13.85) or the iACT group (*F*_1,8_=2.02, *MSE*=15.88, *P*=.19). The Friedman-test using LOCF supported these findings, indicating no significant within-group differences for the iCBT group (*χ* ^2^_1_=3.00,*P*=.08) or the iACT group ( *χ ^2^*_1_=1.00,*P*=.32).

The split-plot ANOVA using CCA yielded constistent results. The main effect was not significant in the iCBT group (*F*_1,2_=2.04,*MSE*=63.17,*P=.29*) or the iACT group (*F*_1,4_=0.27, *MSE*=3.40,*P*=.64). The Friedman-test using CCA confirmed no significant within-group differences for the iCBT group (*χ ^2^*_1_=3.00, *P*=.08) or the iACT group (*χ* ^2^_1_=1.00,*P*=.32).

*GAD-7*

To assess changes in anxiety symptoms, a split-plot ANOVA was conducted for both the iCBT and iACT groups. Results were analyzed using both *Intent-to-Treat* (ITT) with LOCF, and *Complete Case Analysis* (CCA).

In the LOCF analysis, the main effect of time on GAD-7 scores was not significant for the iCBT group (*F*_2,16_=0.57,*P*=.59) or the iACT group (*F*_2,16_=0.42,*P*=.67). Mean scores in the iCBT group decreased slightly from pretreatment (M=8.25,SD=4.98) to posttreatment (M=6.75,SD 4.17). Similarly, iACT scores decreased from pretreatment (M=9.33, SD6.61) to posttreatment (M=7.78, SD 5.07).

The CCA yielded consistent results, with no significant main effect of time for either the iCBT group (*F*_2,6_=0.57,*P*=.59) or the iACT group (*F*_2,8_=0.42,*P*=.67). Non-parametric Friedman tests confirmed these findings, showing no significant within-group differences for iCBT ( *χ*^2^_2_=1.44,*P*=.49) or iACT ( *χ*^2^_2_=0.36,*P*=.83).Kruskal-Wallis tests indicated no statistically significant differences between the iCBT and iACT groups at any time point: pretreatment *P*=.85), midtreatment (*P*=.41), or posttreatment (*P*=.60).

*WHODAS*

To evaluate changes in functional disability, a split-plot ANOVA was performed for both groups using LOCF and CCA.

In the LOCF analysis, the main effect of time on WHODAS scores was not significant for either the iCBT group (*F*_2,16_=0.55,*P*=.58) or the iACT group (*F*_2,16_=0.36,*P*=.45). Friedman tests confirmed this lack of within-group change for both iCBT ( *χ* ^2^_2_=0.78,*P*=.68) and iACT ( *χ* ^2^_2_=1.58,*P*=.45).

The CCA results were consistent with the LOCF findings, showing no significant main effect of time for iCBT (*F*_2,14_=0.24,*P*=.79) or iACT (*F*_2,8_=0.30,*P*=.57). Friedman tests for the CCA also indicated no significant differences ( *χ* ^2^_2_ range: 0.67 to 1.00,*P*>.60). Kruskal-Wallis tests showed no statistically significant differences between groups at pretreatment (*P*=.13), midtreatment (*P*=.19), or posttreatment (*P*=.16).
