# Supplementary material for: Conceptualizing Acceptance and Knowledge as Process Variables in Internet-Delivered and Therapist-Supported Cognitive Behavioral Therapy and Acceptance and Commitment Therapy in Primary Care for Insomnia: Pilot Feasibility and Process-Oriented Randomized Controlled Trial
Source: JMIR Form Res. 2026 May 21;10:e81285. doi: 10.2196/81285 (PMC13193663; doi:10.2196/81285)
Supplement: Multimedia Appendix 4 [file formative-v10-e81285-s004.pdf]

Table S1. Participant attitudes toward treatment at pretreatment

| **Question** | **iCBT (*n*=9)** | **iACT (*n*=9)** |
|---|---|---|
| | Mean (SD) | |
| 1. Have you previously read about the problems for which you have sought this treatment? | 5.44 (2.555) | 4.78 (2.682) |
| 2. How would you rate your knowledge about the problems and treatment for which you have sought this treatment? | 4.33 (1.732) | 4.67 (2.000) |
| 3. How important do you think that knowledge about the problems and treatment is for the treatment to be helpful? | 7.56 (1.333) | 7.11 (1.691) |
| | *n* | |
| 4. a) Do you think that internet therapy seems to be a good wat to acquire knowledge about your problems compared to other sources such as books?<br>- Yes<br>- No<br>- Don't know | <br><br><br>6<br>0<br>3 | <br><br><br>6<br>0<br>3 |
| 4. b) Justify your answer (free-text answer)<br>- Equally good/bad<br>- Internet treatment is accessible<br>- I dislike reading books<br>- More engaging than books | <br>1<br>1<br>0<br>1 | <br>1<br>1<br>1<br>0 |
| 5. Have you previously taken any online courses (e.g. distance learning courses via university or treatment programs via the computer?<br>- Yes<br>- No | <br><br><br>6<br>3 | <br><br><br>5<br>4 |
| 6. Have you had talk therapy before?<br>- Yes<br>- No | <br>4<br>5 | <br>5<br>4 |
| | Mean (SD) | |
| 7. a) How do you think internet therapy compares to talk therapy? | 5.67 (1.118) | 4.78 (0.833) |
| | *n* | |
| 7. b) Justify your answer (free-text answer)<br>- I prefer talk therapy<br>- Online therapy is more flexible<br>- I don't know yet | <br>2<br><br>2 | <br>1<br>2<br>3 |

**Note:** Participants rated items on a Likert scale ranging from 1 to 9, where 1 indicates the lowest level of agreement/satisfaction and 9 indicates the highest.

Abbreviations: iACT: internet-delivered Acceptance and Commitment Therapy; iCBT: internet-delivered Cognitive Behavioral Therapy; ISI: Insomnia Severity Index; LOCF: Last Observation Carried Forward; *M*: mean; *SD*: standard deviation.

Table S2. Participant attitudes toward treatment at posttreatment

| **Question** | **iCBT (*n*=3)** | **iACT (*n*=5)** |
|---|---|---|
| | Mean (SD) | |
| 1. Now that you have completed the treatment. How would you rate your knowledge about the problems and treatment for the problems you have sought this help for? | 6.00 (2.000) | 7.00 (1.871) |
| | *n* | |
| 2. a) Now that you have completed the treatment. Do you think that internet treatment is a good way to acquire knowledge about your problems compared to other sources such as books? (free-text answer)<br>- Yes<br>- No<br>- Unsure | <br><br>3<br>0<br>0 | <br><br>4<br>0<br>1 |
| 2.b) Justify your answer (free-text answer)<br>- Clear instruction and easy to get an overview<br>- Simplicity and accessibility<br>- Easier than books<br>- Blended works well | <br>1<br>0<br>0<br>0 | <br>0<br>1<br>1<br>1 |
| 3.a) How do you think that internet treatment seems to be compared to traditional talk therapy? | 6.00 (2.646) | 5.60 (1.673) |
| | *n* | |
| 3.b) Justify your answer (free-text answer)<br>- It's easier to talk if you see the person<br>- The timing of the exercises is better<br>- Blended works well | <br>0<br>1<br>1 | <br>1<br>1<br>1 |
| | Mean (SD) | |
| 4. a) Has the knowledge you have acquired in this treatment helped you change your view of your problems? | 6.33 (2.517) | 5.00 (2.828) |
| | *n* | |
| 4.b) Justify your answer (free-text answer)<br>- Useful tool for processing and learning<br>- No, I already tried multiple treatments | <br>2<br>0 | <br>1<br>1 |
| | Mean (SD) | |
| 5. How helpful has the knowledge provided in this treatment been to you? | 7.67 (0.577) | 5.00 (2.828) |
| 6. Do you think that what you learned during treatment will help you in the long term? | 7.00 (1.000) | 5.60 (3.286) |
| | *n* | |
| 7. What did you find most helpful about what you learned during treatment? (free-text answer)<br>- Relaxation and sleep restriction<br>- Knowledge on sleep<br>- Sleep planning<br>- Accessibility<br>- Not controlling sleep<br>- Reminder to do the exercises | <br>1<br>1<br>1<br>0<br>0<br>0 | <br>0<br>0<br>0<br>1<br>1<br>1 |

| | | |
|---|---|---|
| - Bodyscan | 0 | 1 |
| 8. How did you experience having contact with the treatments mainly via messages? (free-text answer)<br>- Good<br>- OK<br>- Too much delay | <br><br>2<br>1<br>0 | <br><br>3<br>1<br>1 |
| 9. Overall, what has been good about the treatment? (free-text answer)<br>- Good advice and videos<br>- The holistic approach<br>- Learning about sleep and the mind<br>- Reminder to focus on the exercises<br>- Reduced anxiety | <br><br>1<br>1<br>0<br>0<br>1 | <br><br>1<br>0<br>1<br>1<br>1 |
| 10. Overall, what has been less good about the treatment? (free-text answer)<br>- The contact with the psychologist<br>- Too time-consuming<br>- The Sleep diary<br>- Too many short videos<br>- I would prefer to meet in person<br>- Too much focus on how much I sleep | <br><br>1<br>1<br>0<br>0<br>0<br>0 | <br><br>0<br>0<br>1<br>1<br>1<br>1 |
| 11. Overall, what would you like to have more of in the treatment? (free-text answer)<br>- Dialogue<br>- Time<br>- Keeping the information afterwards<br>- Mindfulness | <br><br>1<br>1<br>0<br>0 | <br><br>0<br>0<br>1<br>2 |
| 12. Overall, what would you like to have less of in the treatment? (free-text answer)<br>- Less text and more exercises<br>- Nothing | <br><br>1<br>2 | <br><br>0<br>4 |

Note: Ratings are based on a 9-point Likert scale ranging from 1 (lowest) to 9 (highest). Results are presented using listwise deletion; only participants who provided data at this time point are included (*n*=3 for iCBT; n=5 for iACT).

Abbreviations: iACT: internet-delivered Acceptance and Commitment Therapy; iCBT: internet-delivered Cognitive Behavioral Therapy; ISI: Insomnia Severity Index; LOCF: Last Observation Carried Forward; *M*: mean; *SD*: standard deviation.
